# DCE-MRI of the Liver: Reconstruction of the Arterial Input Function Using a Low Dose Pre-Bolus Contrast Injection

**DOI:** 10.1371/journal.pone.0115667

**Published:** 2014-12-29

**Authors:** Guido H. Jajamovich, Claudia Calcagno, Hadrien A. Dyvorne, Henry Rusinek, Bachir Taouli

**Affiliations:** 1 Translational and Molecular Imaging Institute, Department of Radiology, Icahn School of Medicine at Mount Sinai, New York, New York, United States of America; 2 Department of Radiology, New York University Langone Medical Center, New York, New York, United States of America; West German Cancer Center, Germany

## Abstract

**Purpose:**

To assess the quality of the arterial input function (AIF) reconstructed using a dedicated pre-bolus low-dose contrast material injection imaged with a high temporal resolution and the resulting estimated liver perfusion parameters.

**Materials and Methods:**

In this IRB–approved prospective study, 24 DCE-MRI examinations were performed in 21 patients with liver disease (M/F 17/4, mean age 56 y). The examination consisted of 1.3 mL and 0.05 mmol/kg of gadobenate dimeglumine for pre-bolus and main bolus acquisitions, respectively. The concentration-curve of the abdominal aorta in the pre-bolus acquisition was used to reconstruct the AIF. AIF quality and shape parameters obtained with pre-bolus and main bolus acquisitions and the resulting estimated hepatic perfusion parameters obtained with a dual-input single compartment model were compared between the 2 methods. Test–retest reproducibility of perfusion parameters were assessed in three patients.

**Results:**

The quality of the pre-bolus AIF curve was significantly better than that of main bolus AIF. Shape parameters peak concentration, area under the time activity curve of gadolinium contrast at 60 s and upslope of pre-bolus AIF were all significantly higher, while full width at half maximum was significantly lower than shape parameters of main bolus AIF. Improved liver perfusion parameter reproducibility was observed using pre-bolus acquisition [coefficient of variation (CV) of 4.2%–38.7% for pre-bolus vs. 12.1–71.4% for main bolus] with the exception of distribution volume (CV of 23.6% for pre-bolus vs. 15.8% for main bolus). The CVs between pre-bolus and main bolus for the perfusion parameters were lower than 14%.

**Conclusion:**

The AIF reconstructed with pre-bolus low dose contrast injection displays better quality and shape parameters and enables improved liver perfusion parameter reproducibility, although the resulting liver perfusion parameters demonstrated no clinically significant differences between pre-bolus and main bolus acquisitions.

## Introduction

DCE-MRI of the liver can be used to quantify liver parenchymal and tumor perfusion by monitoring the uptake and washout of gadolinium based contrast agents injected intravenously. To assure the coverage of entire organs in the abdomen, serial 2D or 3D T1-weighted images are acquired over time. To achieve sufficient spatial resolution and coverage, current DCE-MRI of the liver is typically acquired with a temporal resolution ranging between 3.5 and 7.5 sec [1]–[6]. In addition to extracting tissue activity curves in each voxel or in larger regions (1,2), quantification of tissue perfusion relies on the determination of: 1) the arterial input function (AIF), generally measured in the abdominal aorta, given the small size of branches such as the hepatic or renal artery, 2) the portal vein input function, given the hepatic dual vascular input.

The first pass of the contrast agent bolus in the AIF consists of a rapidly changing, high peak concentration that poses challenges for measuring it [7]. The erroneous determination of the AIF could result in miscalculation of perfusion parameters [8], [9]. Sources of error in the AIF determination include the non-linear relationship between the observed signal intensity (SI) and gadolinium concentration ([Gd]) [10], T2* effects [11], inflow artifact, especially pronounced in axial acquisitions [12], and the misrepresentation of the rapidly changing concentration peak due to insufficient temporal resolution [7], [9], [13].

One option to accurately determine the AIF is to reduce the injected contrast dose. However, this approach leads to lower signal to noise ratio (SNR) in the tissue of interest [14]. Another option consists of using a population based AIF [15], disregarding inter-individual variations (e.g. due to cardiac output differences) in the AIF. However, population based AIF introduces significant bias on the estimated values for the pharmacokinetic parameters, of special concern when dealing with patients who suffer from cardiopulmonary disease.

An attractive alternative is the use of a low dose pre-bolus injection prior to the main DCE-MRI exam. SI is measured in the abdominal aorta after the low-dose injection of contrast agent and the required AIF after the full-dose injection of contrast agent is then reconstructed. This technique was successfully validated in animal studies [16], human heart [17]–[21], lungs [14], [22], breast [23] and brain tissues [24]. Results achieved using a low dose pre-bolus injection are encouraging, generally showing an improvement in perfusion estimation precision over conventional DCE-MRI. However, there is no published data on the use of pre-bolus acquisition in the liver.

The objective of our study was to assess the quality of a reconstructed AIF in the abdominal aorta using a low dose pre-bolus contrast injection compared to the AIF obtained after main bolus injection for liver DCE-MRI and to quantify liver perfusion parameters and parameter reproducibilities using both methods.

## Materials and Methods

### Subjects

This HIPAA compliant single center prospective study was approved by the Icahn School of Medicine at Mount Sinai Program for the Protection of Human Subjects. Written consent was obtained from all patients prior to the exam. The study included 21 subjects (M/F 17/4, mean age 57 y, range 30–67 y) recruited through our local Hepatology clinic. Three subjects underwent repeat DCE-MRI exams to assess test-retest reproducibility of AIF shape and liver perfusion parameters. The second exam took place with a mean delay of 16 days (1, 7 and 41 days between exams). Patients with decreased renal function (eGFR <60 ml/min/1.73 m^2^) were excluded from the study. Since portal venous flow can increase postprandially [25], all subjects were asked to fast for 6 hours before the study. Data was partially reported in meeting proceedings [26].

### DCE-MRI acquisition

**Figure 1:**
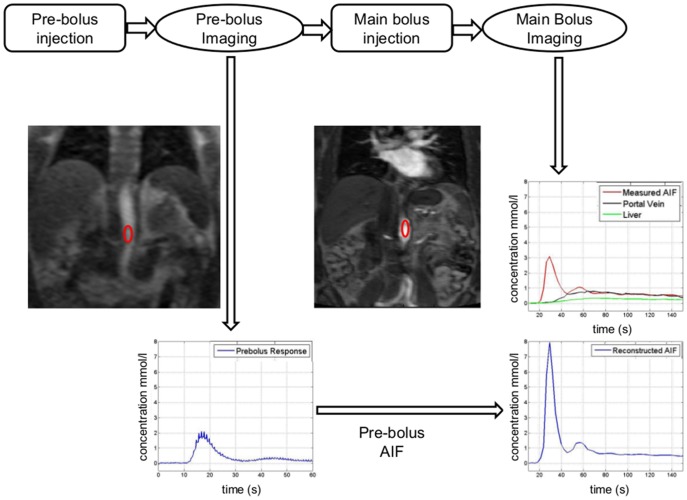
Flow diagram showing pre-bolus and main bolus AIF acquisition and processing for DCE-MRI of the liver in a 67 year-old male patient with HCV. The pre-bolus protocol consists of a high temporal resolution 2D acquisition (0.2 s). An ROI is then placed in the abdominal aorta and the concentration curve is extracted. This curve is used to reconstruct pre-bolus AIF. Main bolus imaging protocol consists of 3D acquisition with higher spatial resolution/lower temporal resolution (3.2 s). ROIs are placed on the abdominal aorta, portal vein and liver parenchyma. Pre-bolus AIF curve (bottom) demonstrates better quality compared to main bolus AIF (top).

All examinations were performed on a 1.5T clinical MRI system (Magnetom Avanto, Siemens AG, Healthcare Sector, Erlangen, Germany) equipped with a multichannel spine and body matrix coil and 45 mT/m maximum gradient strength. Patients were positioned arms up in supine position. The following sequences were acquired (**Fig. 1** **,** **Table 1**):

**Table 1:** Sequence parameters of the Look-Locker for T1 mapping, 2D-TurboFLASH for pre-bolus acquisition and 3D-FLASH sequences for main bolus acquisition for liver DCE-MRI.

	Look-Locker	2D-TurboFLASH	3D-FLASH
**Acquisition Plane**	Axial	Coronal oblique	Coronal
**TR (ms)**	23.49	2.3	2.96
**TE (ms)**	1.12	0.91	0.95
**Flip angle (°)**	10	12	12
**FOV Read (mm)**	430	430	400
**FOV Phase (%)**	75	90.5	100
**Slice thickness (mm)**	10	10	4 (interpolated)
**Acquisition matrix**	192×144	128×93	192×121
**Number of slices**	1	1	36
**Parallel imaging**	1	No	Yes (GRAPPA 3)
**Scan time (min)**	0.3	1	3.4 (3.0–3.8)*
**Temporal resolution (s)**	-	0.2	3.2 (2.6–7.4)*
**Number of volumes**	-	-	64

*average and range.

- Breath-hold axial, coronal and sagittal T2 HASTE sequences to localize the abdominal aorta, portal vein and liver.
- Pre-bolus DCE-MRI: A saturation recovery 2D-TurboFLASH sequence with recovery time of 120 ms was used in a coronal oblique plane and centered on the proximal abdominal aorta. Images were acquired before, during and after the injection of pre-bolus injection of a fixed dose of 1.3 mL of gadobenate dimeglumine (Multihance, Bracco Diagnostics) at 4.1 mL/s followed by a 25 mL saline flush using an MR compatible injector. The selected dose provides SNR in the aorta, which makes the measurement of the arterial response possible. The low dose of contrast agent is assumed to be washed out before the injection of the main bolus. The injection rate was the highest rate allowed by the power injection given the small dose of contrast agent. The pre-bolus was not diluted in order to use the injector without operator manipulation between pre- and main bolus injections. A small flip angle [23] and a coronal plane were selected in order to minimize the inflow effects. Patients were instructed to take a long breath-hold followed by shallow respiration for a total of 60 s.
- T1 mapping: The baseline hepatic T1 value was obtained using a breath-hold Look-Locker sequence [27] before the injection of the main bolus of contrast agent.
- Main bolus DCE-MRI of the liver: Immediately after the pre-bolus injection, a 3D-FLASH sequence was used in the coronal plane to obtain 3 pre-contrast acquisitions and 64 post contrast acquisitions after the injection of the main bolus of contrast agent (0.05 mmol/Kg of gadobenate dimeglumine followed by a 25 mL saline flush injected at a rate of 5 mL/s with an MR-compatible power injector). The injection rate was selected as in [4], [28]. Patients were instructed to take an initial long breath-hold followed by shallow respiration for a total of 60 sec, followed by multiple breath-holds separated by short periods (4 s) of quick breathing.

Additionally, a blood sample was extracted and the hematocrit value was measured.

### Image analysis

Images were processed by observer 1 (---, postdoctoral fellow with 2 years of experience in MR image analysis). Regions of Interest (ROIs) were placed in the abdominal aorta at the level of the celiac axis to measure SI using Osirix (v4.1.1; Pixmeo, Switzerland) (**Fig. 1**). Additional ROIs were placed in the portal vein and liver parenchyma after image coregistration with in-house software implemented in MatLab 2014a (MathWorks, Natick, MA). ROI placements were supervised by a body MR radiologist (observer 2, —) with 10 year' experience. The conversion from SI to contrast agent concentration ([Gd]) was performed by inverting the non-linear relationship given by the SPGR signal equation [27], [29] assuming a pre-contrast T1 value for blood of 1200 ms [30] and obtaining the T1 value for the liver parenchyma from the T1 map. Blood contrast agent concentrations were converted to plasma concentrations using the measured hematocrit value of each patient.

#### Pre-bolus AIF

**Figure 2:**
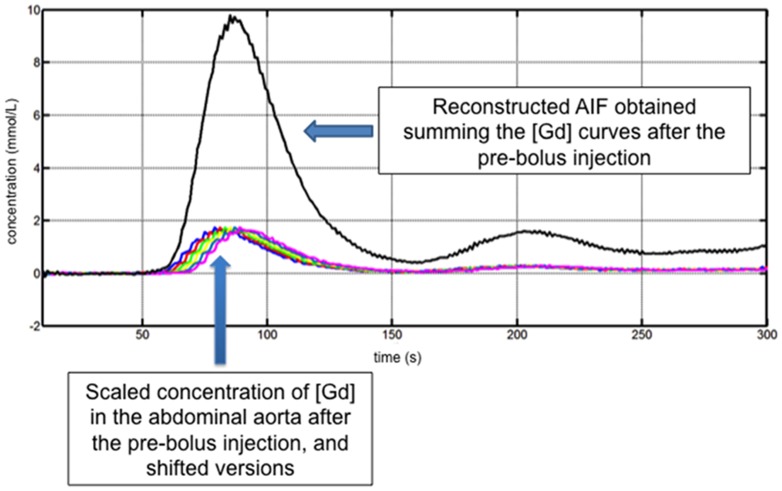
AIF reconstruction using measured gadolinium concentration in the abdominal aorta after pre-bolus injection in a 52 year-old male patient with HCV. The reconstructed AIF consists of the addition of scaled time-shifted versions of the response to low-dose pre-bolus injection of contrast agent. The concentration curve in the abdominal aorta displays rapid oscillations superimposed to the expected signal shape due to in-flow effects, which are minimized by the selection of the small flip angle and a coronal oblique acquisition that contains the abdominal aorta.



From each pre-bolus concentration curve, the pre-bolus AIF was reconstructed by scaling, time-shifting and summing until obtaining an equivalent duration of the injections, as described previously [31] for the same pre-bolus and main bolus injection rates (**Fig. 2**). This AIF reconstruction relies on the assumption that the system formed between the injection and the output observed in the aorta is linear and time-invariant [22]. Then, under the same linear and time-invariant assumptions, for a pre-bolus and main bolus injection rates r_p_ and r_m_ respectively, duration of pre-bolus and main bolus durations T_p_ and T_m_, the pre-bolus concentration AIF C_a_(t) can be reconstructed from the pre-bolus concentration in the aorta C_p_(t) as follows:

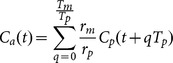
.

This formulation allows for the AIF reconstruction even in cases where the pre-bolus and main bolus injection rates are different. When the injection rates coincide, this reconstruction formula is equivalent to the one used in previous publications [22].

#### Qualitative evaluation of pre-bolus and main bolus AIF curves

An ideal AIF is expected to present a steep upslope, a high concentration first pass peak and a steep downslope, followed by a small recirculation peak [32]. Two observers (—, observer 2, and observer 3, —, a postdoctoral fellow with 7 years experience in perfusion processing) assessed blindly and independently pre-bolus and main bolus AIF curves qualitatively focusing on their shapes, primarily in the first pass peak, the width of this peak, and the presence of a recirculation peak. The observers chose whether the reconstructed or the measured AIF was of better quality.

#### Quantitative evaluation of pre-bolus and main bolus AIF curves

Peak concentration (Cpeak, in mmol/L), time to peak (TTP, in s), upslope (mmol/s.L), area under the time activity curve of gadolinium contrast at 60 s (AUC60, in mmol.s/L) and the full width at half maximum (FWHM, in s) were calculated and compared between pre-bolus and main bolus concentration vs. time AIF curves.

**Figure 3:**
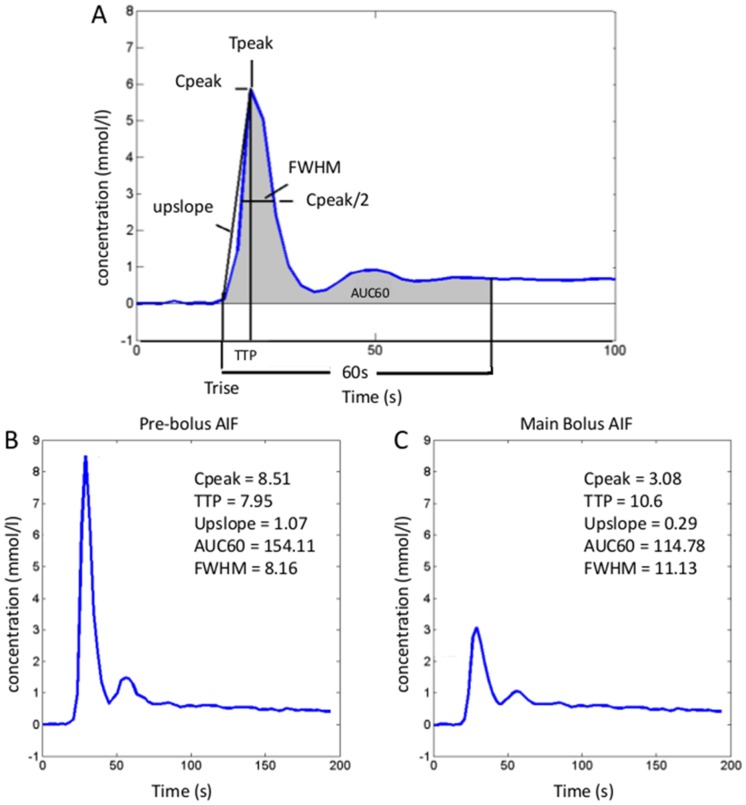
Diagram depicting calculated AIF (arterial input function) parameters (A). An example is shown in a 67-year-old patient with HCV (same patient as in Fig. 1). Pre-bolus AIF (B) and main bolus AIF (C). Pre-bolus AIF demonstrates higher peak concentration, upslope and AUC60, shorter TTP and smaller FWHM (values are given on the figures).

Tpeak was defined as the time point at which the gadolinium concentration [Gd] reached its maximum and Trise as the time point at which the concentration curve exceeds the baseline threshold, then TTP  =  Tpeak-Trise. Cpeak is [Gd] at Tpeak. Upslope is calculated as Cpeak/TTP. AUC60 is the area under [Gd] from to Trise to Trise+60 s. FWHM measures the width of the first pass peak by computing the time lapse from the point where the curve is [Gd] = Cpeak/2, before the peak to the point where the curve is [Gd] = Cpeak/2 after the peak (**Fig. 3**).

#### Modeled Hepatic Perfusion Parameters

Both reconstructed and measured AIF were used in conjunction with the portal vein and liver concentration curves to estimate liver perfusion parameters. Considering the dual blood supply, a dual-input single compartment model [33], [34] can be used to model the liver parenchyma, given by



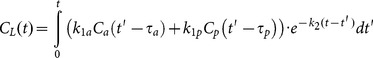
,

where C_a_(t), C_p_(t), and C_L_(t) represent the AIF, the concentrations of contrast agent in the portal vein, and in the liver, respectively; τ_a_ and τ_p_ account for the delay between the AIF and portal vein delays with respect to the liver parenchyma, respectively; and k_1a_ represents the aortic inflow rate constant, k_1p_ the portal venous inflow rate constant, and k_2_ the outflow rate constant. Parameters k_1a_, k_1p_, k_2_, τ_a_ and τ_p_ were estimated by fitting the concentration curves via nonlinear least-squares implemented using MatLab 2014a.

Estimated rates were converted to flow parameters by multiplication by 60 s/min×100 ml/ml [13]: arterial flow (Fa, ml/min/100 ml), portal venous flow (Fp, ml/min/100 ml), total liver blood flow (Ft = Fa+Fp, ml/min/100 ml), arterial fraction (ART = 100*Fa/Ft, %), portal venous fraction (PV = 100*Fp/Ft, %), the distribution volume of Gd-contrast through the liver compartment (DV = 100.(k_1a_+k_1p_)/k_2_, %), and the average time it takes a Gd molecule to traverse the liver from the arterial or portal inputs to the venous output given by the mean transit time (MTT = 1/k_2_, s). These parameters were calculated using both reconstructed and measured AIFs.

### Statistical analysis

MatLab 2014a was used for statistical analysis. The significance of the quality evaluations by the two observers was assessed using the Fisher exact test. Quantitative shape parameters of pre-bolus and main bolus AIFs concentration vs. time curves for every MR exam and the resulting liver perfusion parameters were compared using a paired Wilcoxon test. Differences in perfusion parameters estimated with and without the pre-bolus injection were evaluated by computing the coefficient of variation (CV) and Bland-Altman 95% limits of agreement. Test-retest parameter reproducibilities were evaluated by computing the CV of AIF shape and liver perfusion parameters in 3 patients.

## Results

24 DCE-MRI exams of the liver of all 21 patients were analyzed. Twenty patients had chronic hepatitis C infection and 1 patient had nonalcoholic steatohepatitis. Liver biopsy findings performed within 3 months of the MRI study determined the following fibrosis stages: stage 0 (n = 1), stage 1 (n = 2), stage 2 (n = 9), stage 3 (n = 5) and stage 4 (n = 4).

### Qualitative and qualitative evaluation of AIF

Pre-bolus curve quality was significantly better than that of main bolus AIF curves for both observers. Pre-bolus AIFs demonstrated better curve quality 19/21 and 20/21 times, compared to main bolus AIF, respectively for observer 1 and 2 (p<0.001 for both, Fisher's exact test).

Peak concentration, upslope and AUC60s were all significantly higher for pre-bolus AIF compared to those of the main bolus AIF (p<0.001), while FWHM of pre-bolus AIF was significantly lower compared to FWHM of main bolus AIF (p = 0.006) (**Table 2**). TTP was not significantly different between the 2 acquisitions.

**Table 2:** Quantitative AIF (arterial input function) curve parameters obtained for main bolus and pre-bolus acquisitions (mean ± SD).

	Pre-bolus AIF	Main bolus AIF	p
**Cpeak**	8.14±4.80	3.41±1.54	<0.001
**TTP**	8.77±2.34	9.46±4.87	1.0
**Upslope**	1.00±0.64	0.46±0.31	<0.001
**AUC60**	102.74±27.88	72.11±20.88	<0.001
**FWHM**	8.93±2.60	11.60±4.86	0.006

Cpeak, Upslope and AUC60 for pre-bolus AIF were significantly greater than those of main bolus AIF, while FWHM was significantly smaller. TTP was not different.

Cpeak (peak concentration, in mmol/L), TTP (time to peak, in s), upslope (in mmol/(L.s), AUC60 (area under the time activity curve of gadolinium contrast at 60 sec, in mmol.s/L), FWHM (full width at half maximum, in s).

### Estimated liver perfusion parameters

Only the hepatic arterial blood flow, arterial fraction and portal venous fraction were significantly different when using the pre-bolus AIF (p<0.05). Despite the statistical differences, there was good to excellent reproducibilities between parameters computed with or without the pre-bolus injection (CV<14%, **Table 3**), indicating no clinically significant differences. All other perfusion parameters were not significantly different (p>0.28).

**Table 3:** Quantitative liver perfusion parameters obtained for main bolus and pre-bolus acquisitions (mean ± SD), the coefficients of variation (CV, %) and the Bland-Altman (BA) limits of agreements (%).

	Pre-bolus AIF	Main bolus AIF	Mean CV (%)	BA limits of agreement (%)
**Fa**	54.78±39.98	59.76±39.25	12.83	−25.28, 42.66
**Fp**	439.80±189.67	431.52±183.65	1.54	−13.75, 9.95
**Ft**	494.58±197.55	491.28±194.10	0.91	−7.42, 6.08
**ART**	12.54±10.71	13.59±10.31	13.24	−19.36, 35.40
**PV**	87.46±10.71	86.41±10.31	0.85	−5.32, 2.91
**DV**	72.13±18.54	71.90±18.65	0.44	−3.04, 2.41
**MTT**	18.89±9.47	18.91±9.50	0.70	−2.69, 2.88

Fa (hepatic arterial blood flow, ml/100 ml/min); Fp (hepatic portal blood flow, ml/100 ml/min); Ft (total hepatic blood flow, ml/100 ml/min); ART (arterial fraction, %); PV (portal venous fraction, %); DV (distribution volume, %); MTT (mean transit time, s).

### Reproducibility of AIF shape and liver perfusion parameters (Table 4)

In the 3 patients who underwent test-retest studies, all quantitative AIF shape parameters and liver perfusion parameters except for distribution volume showed better reproducibility using the pre-bolus AIF.

**Table 4:** Test-retest reproducibility of pre-bolus and main bolus AIF (arterial input function) shape and corresponding hepatic perfusion parameters measured in 3 patients expressed as mean and range of coefficients of variation (in %).

AIF shape parameters	Pre-bolus	Main bolus
**Cpeak**	23.7 (3.1–38.0)	41.2 (10.6–60.7)
**TTP**	5.0 (0.0–20.2)	12.12 (0.0–28.2)
**Upslope**	28.8 (23.3–38.0)	51.3 (10.6–76.2)
**AUC60**	7.8 (2.5–17.3)	24.9 (7.0–35.8)
**FWHM**	12.0 (0.3–29.6)	23.1 (5.7–46.7)

Liver perfusion parameters	Pre-bolus	Main bolus
**Fa**	27.51 (14.14–43.63)	58.42 (9.64–104.50)
**Fp**	38.74 (23.48–47.89)	63.78 (21.49–123.57)
**Ft**	34.93 (23.49–44.80)	43.97 (22.98–73.23)
**ART**	17.32 (1.30–49.35)	71.38 (41.02–128.55)
**PV**	4.19 (0.01–12.49)	35.00 (1.53–91.93)
**DV**	23.57 (0.03–42.92)	15.79 (0.05–43.29)
**MTT**	38.44 (18.16–73.64)	57.46 (22.93–76.13)

Pre-bolus AIF shape parameters demonstrate better reproducibility for all considered parameters. Liver perfusion parameters demonstrate better reproducibility for all considered parameters when using the pre-bolus technique with the exception of distribution volume.

Cpeak (peak concentration), TTP (time to peak), upslope, AUC60 (area under the time activity curve of gadolinium contrast at 60 sec), FWHM (full width at half maximum), Fa (hepatic arterial blood flow), Fp (hepatic portal blood flow), Ft (total hepatic blood flow), ART (arterial fraction), PV (portal venous fraction), DV (distribution volume), and MTT (mean transit time).

## Discussion

The objective of this study was to show that the AIF could be reliably reconstructed by measuring the response to a low dose (1.3 ml) pre-bolus injection of gadolinium contrast for DCE-MRI of the liver. This pre-bolus AIF was compared with the AIF measured after the injection of the main bolus of contrast (0.05 mmol/Kg) and was observed to have better curve quality and better shape features (higher upslope, peak contrast agent concentration, and lower FWHM). AIF shape and resulting liver perfusion parameters show better test-retest reproducibilities (with the exception of distribution volume) when using the pre-bolus AIF. However, the differences in liver perfusion parameters were smaller than test-retest reproducibility, indicating no clinically significant differences.

The AIF measured after the injection of the main dose of contrast agent suffers from deleterious effects such as low temporal resolution and the nonlinear relationship between SI and contrast agent concentration mainly affecting the first pass of the contrast agent [8]. This makes the use of alternative methods of estimating the AIF important for quantifying modeled perfusion parameters. Our results indicate that the pre-bolus AIF can overcome the low temporal resolution and saturation effects observed with the main bolus AIF by taking advantage of the higher temporal resolution acquisition and the lower dose used for pre-bolus (which resides in the linear regime between concentration and observed SI in the acquired MR images).

One technique to correct for the saturation effect after the images were acquired is to convert the SI curves to concentration curves by using the SPGR equation [10], [27]. However, this technique does not correct for low temporal resolution acquisition and the resulting liver perfusion parameters show poor to acceptable reproducibility [27]. The use of lower concentrations of contrast agent as in the pre-bolus injection is shown in this study to be a method that avoids deleterious effects associated with the high-concentration of the main bolus.

Makkat et al [23] observed that the main bolus AIF was undergoing saturation effects in an ROI in the aorta and that a pre-bolus injection could be used instead for breast DCE-MRI. In our study, we employed two different imaging protocols, each tailored for the pre-bolus and main bolus acquisitions separately, in order to achieve a higher temporal resolution for the pre-bolus AIF and a higher spatial resolution after the main bolus injection, as opposed to the study by Makkat et al [23] where the same image acquisition parameters are used for pre and main bolus acquisitions.

Most estimated liver perfusion parameters values found in this study are similar to previously published values of perfusion parameters calculated with DCE-MRI [2], [3], [13], [27], [28] when using both the pre-bolus and measured AIFs. Parameters mean transit time and distribution volume were not significantly different when using the pre-bolus AIF. These two parameters have been shown to be useful when differentiating normal liver from cirrhotic patients [2], [28]. Parameters Fa and ART have also been shown to be predictors of advanced liver fibrosis [28] and of utility to characterize hepatocellular carcinomas and their response to treatment [35], so an accurate method to determine these parameters is necessary.

There are several disadvantages to the pre-bolus technique. The addition of the pre-bolus injection in the abdominal DCE-MRI exams involves a slightly higher dose of contrast agent, extends the duration of the protocol and makes it more complex. A single slice 2D acquisition containing the abdominal aorta needs to be selected in order to achieve a high temporal resolution image acquisition after the low-dose injection of contrast agent. The resulting images suffer from inflow effects, resulting in an apparent decrease of the longitudinal relaxation time of blood flow and rapid oscillations can be seen superimposed to the signal in the abdominal aorta. A small flip angle and coronal acquisition were selected in order to minimize these inflow effects. In addition, the AIF reconstruction process of shifting and summing the acquired pre-bolus signal has a smoothing effect and no oscillations are seen in the final pre-bolus AIF.

New imaging sequences that can achieve higher temporal and spatial resolution using high field systems may possibly make the pre-bolus protocol unnecessary. Examples of such sequences include radial [36], spiral [37] and keyhole [38] acquisitions, which need validation for abdominal DCE-MRI. However, the limited temporal resolution is still a problem under investigation (e.g., [13]) and saturation effects observed in the AIFs are not solved.

The main limitation encountered in this study was the absence of a gold standard for perfusion quantification to assess which AIF is better. However, by having patients undergoing the DCE-MRI exam in multiple occasions, we were able to assess the precisions associated with the pre-bolus and main bolus AIFs. The test-retest reproducibilities found in this study are concordant to those found by our group in a previous study with an independent cohort of six patients undergoing two DCE-MRIs [27]. Another limitation is the small number of patients that were enrolled in this study which precluded assessing the role of low dose pre-bolus AIF in detecting pathology, such as advanced liver fibrosis and cirrhosis. However, this was not the objective of the study.

In conclusion, the reconstruction of the AIF using a high temporal resolution acquisition with a low dose pre-bolus injection of gadolinium contrast has the potential to overcome the low temporal resolution and high-concentration deleterious effects shown in the AIF measured after main bolus injection. The pre-bolus protocol decouples the imaging protocol for the AIF and the organ under investigation, allowing for a higher coverage and higher spatial resolution imaging after main bolus injection. Even though AIF shape and most resulting liver perfusion parameters show better test-retest reproducibilities, we believe that the small differences observed in the liver perfusion parameters and the complexity of the pre-bolus protocol limit the utility of this technique for DCE-MRI of the liver. However, it remains to be seen whether this technique improves perfusion quantification in liver lesions.
